# Return to work after aneurysmal subarachnoid hemorrhage

**DOI:** 10.3389/fneur.2024.1401493

**Published:** 2024-04-30

**Authors:** Angelika Sorteberg, Aslan Lashkarivand, Elin Western

**Affiliations:** ^1^Department of Neurosurgery, Division of Clinical Neuroscience, Oslo University Hospital, Oslo, Norway; ^2^Faculty of Medicine, Institute of Clinical Medicine, University of Oslo, Oslo, Norway; ^3^Department of Physical Medicine and Rehabilitation, Division of Clinical Neuroscience, Oslo University Hospital, Oslo, Norway

**Keywords:** return to work, aneurysmal subarachnoid hemorrhage, fatigue, outcome, poor grade aneurysmal SAH, aneurysm repair, long term follow-up

## Abstract

**Introduction:**

Survivors of aneurysmal subarachnoid hemorrhage (aSAH) often recover without severe physical or cognitive deficits. However, strikingly low levels of engagement in productive employment have also been reported in aSAH patients with good or excellent outcomes. Knowledge about return to work (RTW) after aSAH and predictors of no RTW remain limited and controversial. The study aimed to delineate the return to maximum work capacity up to 5 years after the ictus in a larger number of consecutive aSAH patients from the entire aSAH severity spectrum and to identify demographic and medical predictors of no RTW.

**Methods:**

Data were acquired from a prospective institutional database. We included all 500 aSAH survivors aged > 18 years who were treated between January 2012 and March 2018. In addition to gathering data on work status and the type of work at ictus, we retrieved demographical data and assessed aSAH severity based on the quantification of subarachnoid, intraventricular, and intraparenchymal blood (ICH), as well as the World Federation of Neurological Societies (WFNS) grade. We registered the mode of aneurysm repair (endovascular or surgical) and recorded complications such as vasospasm, newly acquired cerebral infarctions, and chronic hydrocephalus.

**Results:**

Furthermore, work status and the grade of fatigue at follow-up were registered. RTW was assessed among 299 patients who were employed at ictus. Among them, 63.2% were women, and their age was 51.3 ± 9.4 (20–71) years. Return to gainful employment was 51.2%, with complete RTW accounting for 32.4%. The independent predictors of no RTW at ictus were age, the WFNS grade 3, and active smoking. The strongest independent predictor was the presence of clinically significant fatigue, which increased the risk of not returning to work by 5-fold. The chance to return to gainful employment significantly increased with the individual's years of education prior to their hemorrhage. The mode of aneurysm repair was not relevant with regard to RTW. Patients in the WFNS grades 1–2 more often returned to work than those in the WFNS grades 3–5, but our results indicate that neurological motor deficits are linked closer to no RTW than aSAH severity *per se*.

**Conclusion:**

Fatigue needs to be addressed as an important element on the path to return to work integration.

## 1 Introduction

Aneurysmal subarachnoid hemorrhage (aSAH) is a severe type of intracranial bleed with a high rate of mortality and morbidity. Functional outcome after aSAH is often deemed by the presence or absence of neurological deficits, and there exists a paradox in that survivors of aSAH often recover to being physically intact (1–3). However, even without neurological deficits, many suffer from the post-aSAH syndrome, which comprises emotional problems such as depression and anxiety, reduced cognitive function, reduced quality of life, and fatigue (4–11). Commonly used scoring tools for outcomes after aSAH, such as the modified Rankin Score (12), do not completely fathom these sequels (13). Reintegration into social activities and, most importantly, gainful employment may be a more sensitive marker of the true outcome after aSAH. Hence, among patients with a modified Rankin Score of 0 (excellent outcome), only nearly one-third had returned to work, whereas more than half of those with a modified Rankin Score of 1–2 were unemployed even after 1 year of acquired aSAH (14).

The return to work (RTW) after aSAH has been described as exceptionally low, even among patients without obvious physical or cognitive problems (2). No more than half of the independent survivors fully returned to their pre-ictal employment status (3, 5, 15). Patients who suffer an aSAH are, on average, younger than the general stroke population so that many are not only active in work-life but also often are at the peak of their careers. Without severe deficits, the chance to resume work should, therefore, be good. Failure to RTW has a high socioeconomic impact (16). Therefore, it is important to identify predisposing factors in order to address the challenge and facilitate focused rehabilitation efforts. Knowledge about predictors for RTW, however, is still limited and controversial, though there appears to be a consensus that cognitive and emotional problems are linked to the inability to RTW (4, 7, 14, 17–21). Recently, fatigue has been increasingly acknowledged as a frequent, often long-lasting sequel even in good outcome aSAH patients, and there is an interrelation between fatigue, emotional problems, and, to some extent, cognitive performance (22, 23). However, to date, the relation of fatigue to RTW remains poorly elucidated. Clinical variables from the acute phase of the hemorrhage were found to be non-predictive of reduced work capacity after aSAH (24). Furthermore, despite huge advancements in aneurysm repair and neurointensive care from the pre-endovascular treatment era in the 1980ies in which late aneurysm repair (after the vasospasm phase) was practiced, RTW seems not to have improved (14, 25, 26). This finding suggests that treatment-related factors may not be crucial or decisive in the ability to RTW.

Controversial findings in the literature regarding RTW after aSAH may be due to several factors, such as small numbers of included patients and relatively short follow-up periods. Many studies evaluated RTW 6–12 months after the ictus. Considering the ongoing recovery of physical and cognitive performance from 6 months to beyond 1 year after the ictus, this time frame may be too short to truly reflect the final maximum work capacity of an individual (1). This would be especially valid in countries that provide back-to-work plans/employment support extending beyond 12 months. In Norway, patients can receive sickness benefits equal to a full salary for a maximum of 1 year. If the patient's ability to work is impaired by at least 50% beyond this time period, a work assessment allowance (typically 66% of income) can be granted, which, until 2018, could last up to 4 years. The few larger long-term follow-up studies included mainly surgically treated patients and those that did not stratify in accordance with the mode of aneurysm repair and/or aSAH severity (20, 27, 28). A selection bias will also be a natural limitation of studies based on self-report questionnaires, as they will solely include those fit enough to answer questionnaires themselves or even only include those living at home (29). Employment is the result of many interwoven factors that, besides health-related issues, also comprise job opportunities, type of pre-ictal work performed, access to work reintegration programs and welfare benefits, debilitating fatigue, and socioeconomic and personal factors (1, 30–32). These aspects are more difficult to precisely assess, and they are hence not too well-explored, especially considering their interrelation with aSAH-relevant medical variables.

The present study aimed to delineate the return to maximum work capacity within the time frame of work assessment allowance, i.e., up to 5 years after the ictus, stratified by aSAH severity in a larger number of consecutive aSAH patients who were treated in the modern era. We also intended to identify predictors of no RTW, considering demographic data, medical variables from the acute phase of the aSAH, educational status, the amount and type of pre-ictal employment, and chronic post-aSAH fatigue.

## 2 Material and methods

This study is a part of a quality project on treatment and outcome in aSAH patients. It is a retrospective study that retrieved anonymized, prospectively collected data from our institutional quality registry including aSAH patients admitted between January 2012 and March 2018. The quality project has been approved by the hospital's data protection officer (21/11433). Consent from participants was waived due to the nature of the study.

We included aSAH patients that were alive and ≥18 years old in March 2018.

### 2.1 Variables

We retrieved demographic data and occupational status at ictus as well as at the last follow-up. Work status at ictus and follow-up was registered as full-time or part-time percentage employment, permanent disability benefit, retirement, sick leave including work allowance assessment, or status as a student. We also registered years of education. The highest level of work capacity was scored. We registered the time from hemorrhage to when the highest known level of work was reached. The type of work was registered and assigned into one of the three sectors: (1) the health and social sector; (2) the manufacture, construction, farming, and retail sector; and (3) the office, teaching, art, and finance sector. The level of education was scored in years. ASAH severity was expressed in terms of the World Federation of Neurological Societies (WFNS) grade (33). We registered the amount of subarachnoid blood with the modified Fisher score (34), the maximum size of intraparenchymal blood (ICH), and the amount of intraventricular blood using the LeRoux score (35). We also registered any rebleed prior to aneurysm repair, the mode of aneurysm repair, and complications such as vasospasm [the highest degree of vasospasm diagnosed radiologically or by transcranial Doppler ultrasonography as well as clinically symptomatic vasospasm (36)], radiologically documented delayed cerebral ischemia (DCI), and chronic post-hemorrhagic hydrocephalus (the insertion of a permanent cerebrospinal fluid shunt). The length of mechanical ventilatory support and length of stay (LOS) were also retrieved from the database. In addition, we retrieved smoking status at ictus and fatigue at follow-up. Fatigue was scored using the Fatigue Severity Scale (FSS) mean score (37). The FSS is a nine-item questionnaire measuring the impact of fatigue on daily life. The nine statements in the questionnaire were scored on a Likert scale in the range from one to seven, where a higher score indicates larger agreement with the given statement and a larger symptom burden regarding fatigue. An FSS mean score of ≥4 is considered to indicate clinically significant fatigue (37). Patients answered the FSS questionnaire during planned outpatient visits or through a structured telephone interview.

### 2.2 Statistics

Statistical analysis was performed in SPSS v 28 (IBM Corp, Armonk, NY). Categorical variables were presented as frequencies or percentages, and continuous variables were presented as mean and standard deviation (SD) or as median and interquartile range (IQR). No imputation for missing data was performed. The WFNS groups were compared using the Chi-square test for categorical data, a one-way ANOVA for continuous normally distributed data, or the Kruskal-Wallis test for continuous and not normally distributed data. We performed binary logistic univariate regression analysis for salient variables with no RTW as the dependent variable. The variables with a *p*-value of < 0.100 were entered into the multivariate regression analysis with step-wise backward logistic elimination. The *p*-values were presented two-sided and considered significant if the *p*-value was < 0.05.

## 3 Results

### 3.1 Patients

**Figure 1:**
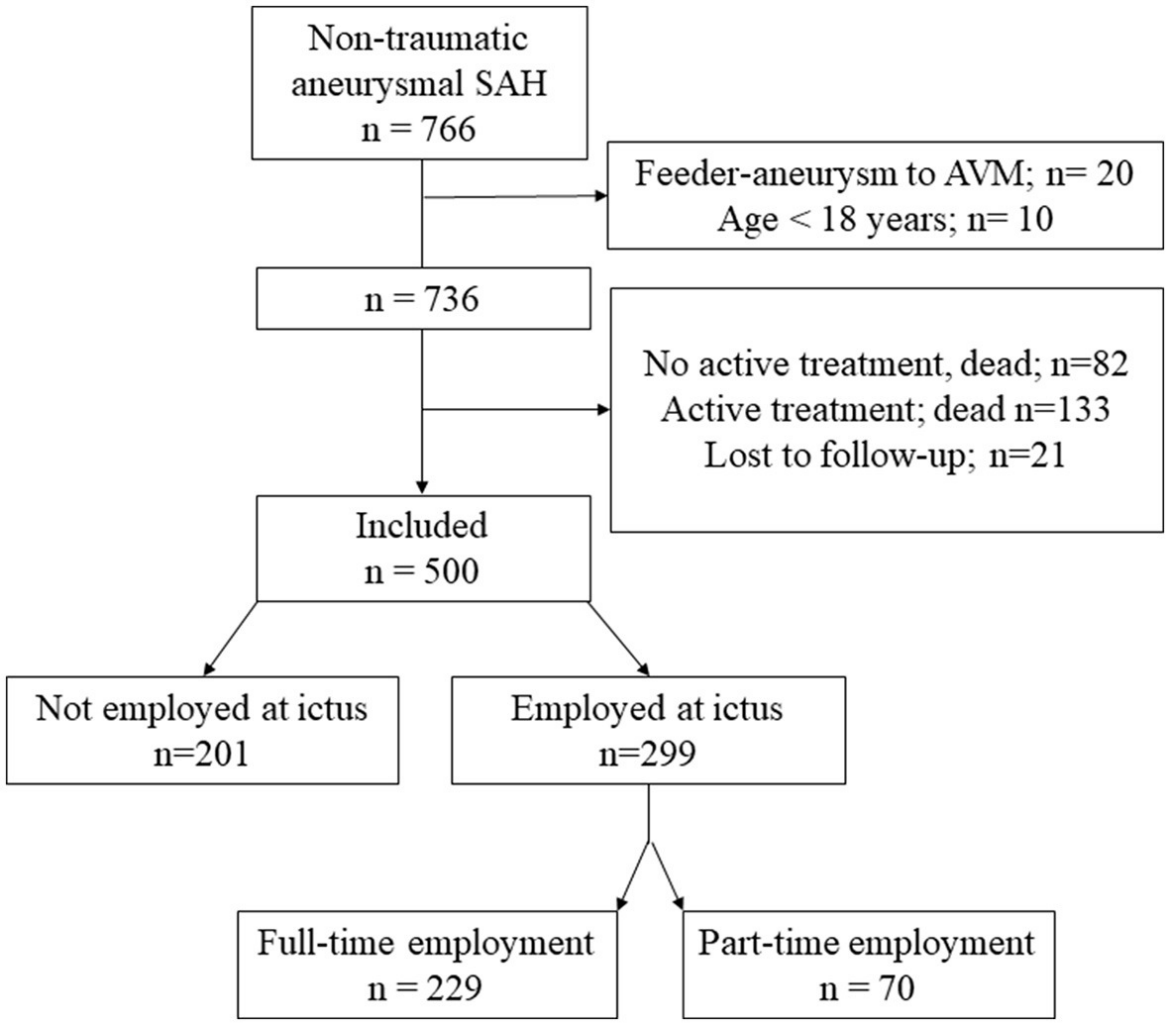
A flowchart of included patients. AVM, arterio-venous malformation; SAH, subarachnoid hemorrhage.

Figure 1 shows the flowchart of included patients. A total of 500 patients were included (with 68.6% being women), and their age was 56.6 ± 12.8 (15–86) years at ictus. Of these, 299 were employed at the time of ictus, 229 were employed full-time, and 70 were employed part-time. Regarding RTW, only the 299 patients who were employed at the time of hemorrhage were included. Complete RTW was achieved by 32.4% of patients and partial RTW was achieved by 18.7% of patients. Return to some gainful employment was achieved by 51.1% of patients.

### 3.2 Demographic and employment status stratified by the WFNS grades

#### 3.2.1 All patients

Table 1 shows demographic data and employment status prior to the hemorrhage and at follow-up for all patients stratified by WFNS grades.

**Table 1:** Demographic data and employment status of all patients.

	**WFNS1**	**WFNS2**	**WFNS3**	**WFNS4**	**WFNS5**	***p* =**
	***n*** = **204**	***n*** = **104**	***n*** = **33**	***n*** = **86**	***n*** = **73**	
Women (%)	63.7	70.2	81.8	70.9	71.2	0.24
Age at ictus, years	55.1 ± 12.9	55.5 ± 13.6	56.3 ± 14.9	60.0 ± 11.8	58.3 ± 10.8	0.039
	(15–84)	(16–86)	(21–82)	(31–84)	(32–86)	
Years to follow-up, median, IQR	3.1 (1.5; 5.3)	2.7 (1.0; 4.6)	2.3 (1.0; 5.4)	2.3 (1.0; 4.4)	2.0 (1.0; 4.4)	0.016
Education, years	12.6 ± 2.7	11.8 ± 2.8	11.7 ± 2.4	12.1 ± 2.8	12.3 ± 2.6	0.273
Fatigue Severity Scale (FSS) mean score	4.40 ± 1.80	4.94 ± 1.58	4.49 ± 1.90	4.97 ± 1.53	5.01 ± 1.36	0.094
FSS ≥ 4.00 (%)	61.7	65.6	53.6	76.5	73.6	0.085
**Work status at ictus (%)**						
Fulltime employment	52.5	42.3	48.5	33.7	46.6	0.066
Part-time employment	11.8	17.3	9.1	15.1	15.1	0.684
Retired	16.7	19.2	30.3	31.4	26	0.036
Disability benefit	14.7	14.4	6.1	16.3	9.6	0.494
Student, pupil	1.5	1	3	–	–	0.487
Homemaker	–	2.9	–	1.2	1.4	0.181
Sick leave/work assessment allowance	2.9	2.9	3	2.3	1.4	0.962
**Work status at follow-up (%)**						
Fulltime employment	23	18.3	9.1	8.1	9.6	0.005
Part-time employment	20.1	10.6	–	9.3	15.1	0.007
Retired	20.6	25	39.4	33.7	30.1	0.052
Disability benefit	31.9	34.6	39.4	43	38.4	0.436
Student, pupil	1	1.9	3	–	–	0.423
Homemaker	0.5	2.9	–	–	–	–
Sick leave/work assessment allowance	2.9	6.7	9.1	5.8	6.8	0.394

Patients in WFNS 4 were older than those in WFNS 1 (*p* = 0.003) and WFNS 2 (*p* = 0.017), and hence, there were more retired individuals in WFNS grades 3–5 than in WFNS grades 1–2 (*p* = 0.002). Follow-up time was longer in WFNS 1 patients than in WFNS 4 (*p* = 0.018) and WFNS 5 patients (*p* = 0.006). The FSS mean score and the proportion of clinically significant fatigue score were lower in WFNS 1 patients than in WFNS 2 (*p* = 0.024), WFNS 4 (*p* = 0.036), and WFNS 5 patients (*p* = 0.039).

#### 3.2.2 Patients who were employed at the time of hemorrhage

Among 299 patients who were employed at the time of hemorrhage, 63.2% were female patients, and their age was 51.3 ± 9.4 (20–71) years. Female participants had higher education levels than male participants (12.9 ± 2.6 years vs. 12.1 ± 2.3 years, *p* = 0.016). Among all employed patients, 87.3% were younger than 62 years (62 years being the lowest possible age for retirement), 40.1% were younger than 50 years, and 11.4% were younger than 40 years. Their follow-up time was a median of 3.3 years (1.5; 5.3 years). Table 2 shows their demographic and employment data stratified into WFNS grades. There were no significant differences in gender distribution, age, length of education, follow-up time, or FSS between categories of WFNS.

**Table 2:** Demographic and employment data stratified into the WFNS grade for those who were employed at the time of hemorrhage.

	**WFNS 1**	**WFNS 2**	**WFNS 3**	**WFNS 4**	**WFNS 5**	***p* =**
	***n*** = **131**	***n*** = **62**	***n*** = **19**	***n*** = **42**	***n*** = **45**	
Women (%)	61.8	62.9	84.2	57.1	64.4	0.354
Age at ictus, years	50.8 ± 10.0	50.1 ± 10.1	52.8 ± 9.9	52.2 ± 8.5	52.7 ± 7.8	0.761
	(25–71)	(20–68)	(36–67)	(31–67)	(32–66)	
Years to follow-up	3.4(1.5;5.4)	3.3(1.5;5.4)	2.5(1.1;5.7)	3.3(1.9;4.9)	2.4(1.2;4.5)	0.668
FSS mean score	4.42 ± 1.88	4.98 ± 1.54	5.20 ± 1.55	5.05 ± 1.68	4.81 ± 1.42	0.181
FSS ≥ 4.00 (%)	63	71.2	76.5	76.3	73	0.418
Education, years	13.0 ± 2.5	12.5 ± 2.5	11.5 ± 2.4	12.2 ± 2.5	12.4 ± 2.6	0.201
**Employment at ictus (%)**						
Full-time	81.7	71	84.2	69	75.6	0.35
Part-time	18.3	29	15.8	31	24.4	0.35
Average % part-time	60.7 ± 17.6	64.1 ± 17.0	56.7 ± 20.8	51.6 ± 16.4	64.0 ± 18.2	0.332
**The type of work at ictus (%)**						
Health and social sector	21.4	22.6	31.6	14.3	33.3	0.24
Manufacture, construction, farming, and retail sector	35.1	41.9	26.3	54.8	33.3	0.119
Office, teaching, art, and finance sector	43.5	35.5	42.1	31	33.3	0.517
**Work status at follow-up %**						
Full-time	35.1	30.6	15.8	16.7	15.6	0.025
Part-time	31.3	17.7	–	19	24.4	0.019
Average % part-time	50.6 ± 22.3	48.2 ± 29.3	–	45.4 ± 27.8	43.2 ± 20.2	0.784
Retirement	5.3	8.1	15.8	4.8	6.7	0.503
Disability benefit	24.4	32.3	52.6	50	42.2	0.006
Student, pupil	–	1.6	–	–	–	–
Sick leave/work allowance assessment	3.8	9.7	15.8	9.5	11.1	0.123
**RTW (%)**						
Full RTW	43.5	35.5	15.8	19	15.6	< 0.001
Partial RTW	22.9	12.9	–	16.7	24.4	0.08
No RTW	33.6	51.6	84.2	64.3	60	< 0.001
Months until return to work	13.7 ± 12.5	12.3 ± 8.9	9.3 ± 0.6	15.1 ± 10.0	16.1 ± 12.3	0.759
**No RTW within the sector (%)**						
Health and social sector	35.7	50	83.3	66.7	73.3	0.073
Manufacture, construction, farming, and retail	43.5	61.5	100	65.2	66.7	0.071
Office, teaching, art, and finance	24.6	40.9	75	61.5	40	0.016
	*p* = 0.125	*p* = 0.359	*p* = 0.484	*p* = 0.967	*p* = 0.069	

FSS, Fatigue Severity Scale; RTW, return to work.

**Figure 2:**
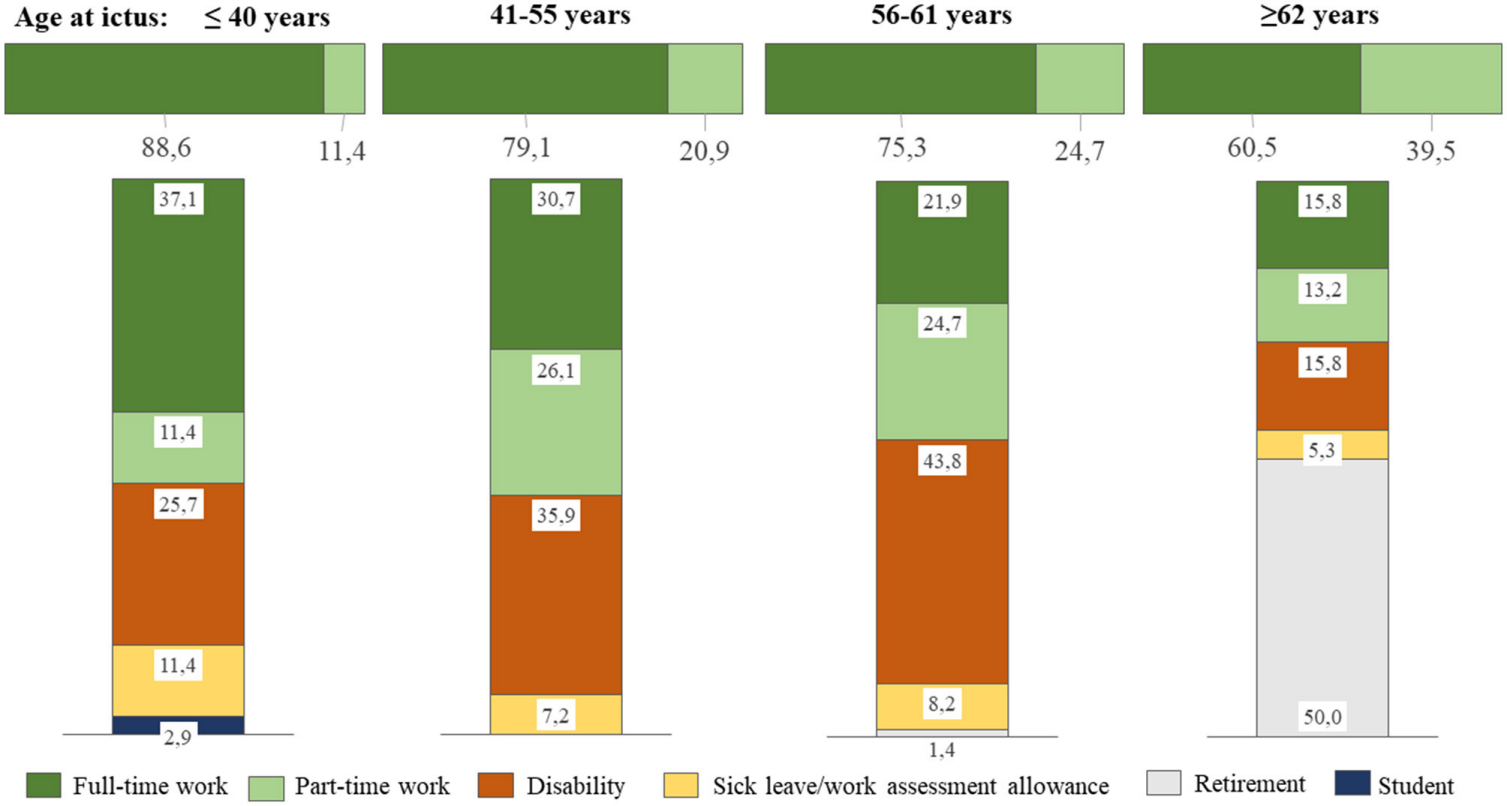
Employment status stratified by age categories. The upper, horizontal bar illustrates the pre-ictal distribution of full-time and part-time employment, whereas the vertical bars show employment status at the last follow-up.

The pre-ictal employment status was full-time in 76.6% of patients and part-time in 23.4% of patients, with the average percentage of part-time employment being 60.1 ± 17.5. The fraction of part-time employment increased with age, as shown in Figure 2, where the upper, horizontal bar illustrates the pre-ictal distribution of full-time and part-time employment. There were no significant differences in pre-ictal employment statuses between WFNS grades. The proportion of those who worked in the manufacturing, construction, farming, and retail sectors was the same as those working in office, teaching, arts, and finance sectors (38.5%), whereas 23.1% worked in the health and social sectors. Patients in WFNS 4 more often worked within the manufacture, construction, farming, and retail sectors than those in WFNS 1 (*p* = 0.024), WFNS 3 (*p* = 0.040), and WFNS 5 (*p* = 0.045). Correspondingly, fewer had worked in the health and social sectors prior to hemorrhage in WFNS 4; this difference reached significance only in comparison to WFNS 5 patients (*p* = 0.038).

At follow-up, full-time employment had significantly decreased from 76.6 to 27.4% (*p* < 0.001), whereas part-time had a non-significant decrease to 14.2%, and the average percentage of part-time employment had decreased from 60.1 ± 17.5 to 48.4 ± 23.5 (*p* < 0.001) for all patients combined. Figure 2 shows employment status at the latest follow-up, stratified by age categories. Returning to full-time employment was more likely to occur when the patient was younger, whereas those ≥62 years were more likely to retire. The rate of those with permanent disability benefits increased with age categories up to 62 years.

The drop in full-time employment was highly significant (*p* < 0.001) for all WFNS grades, but the return to full-time employment at follow-up was approximately twice as high in WFNS 1 and 2 patients as compared to WFNS 3–5 patients (*p* = 0.001), in whom it ranged from 15.6 to 16.7% (Table 2). Part-time employment after the hemorrhage almost doubled in WFNS 1 patients (*p* = 0.11) and remained unchanged in WFNS 5 patients, whereas it dropped in WFNS 2–4 patients. In total, 34.4% of female patients recovered to RTW compared to 29.1% of male patients, which was a non-significant difference.

The largest shift from employment at ictus occurred to disability benefit at follow-up, accounting for 34.1% of all 299 patients. The rate of those receiving disability benefits was significantly different among categories of WFNS grades, with those in WFNS 3–5 grades receiving it more often than those in WFNS 1–2 grades (47.2 vs. 26.9%, *p* < 0.001). At follow-up, 6.7% of patients were retired, and all of them were ≥ 61 years at the time of ictus. There was no difference in retirement between WFNS grades. At follow-up, 7.7% of patients were still on sick leave or work assessment allowance (which was < 4 years past the ictus in all of them). Two of them were 62 years old, but the follow-up time was only 1 year in both patients.

The median time until the maximum working capacity was reached was 11 months (IQR 8.0; 14.0). There were no significant differences in time until employment was maximally resumed between patients in the various WFNS grades, apart from the few WFNS 3 patients who resumed work earlier than WFNS 1 and 5 patients (Table 2). Full RTW was significantly lower in WFNS grades 3–5 (15.6–19.0%) than in WFNS grades 1–2 (44.4 and 35.5%, *p* < 0.001), Table 2. None of the WFNS 3 patients made a partial RTW; apart from this, partial RTW was not significantly different between WFNS grades.

In total, 10 of the 97 patients with full RTW had increased their level of activity (10.3%). They all had low-grade aSAH (5 in WFNS 1 and 5 in WFNS 2), nine out of 10 participants were women, and they were younger than the average cohort (median 46.5 years; 40.0–60.0 years). Seven patients increased from part-time work to full-time employment, whereas the remaining three patients increased their part-time percentage. For all 10 patients, the median increase in work was 25.0% (10–60%). The median time until RTW was 10 months (2–78 months).

No RTW differed significantly between the employment sectors and was the lowest for those who had worked within the office, teaching, art, and finance sector (37.4%, *p* = 0.007). For the health and social sector, the percentage of no RTW was 53.6%, and for the manufacture, construction, farming, and retail sector, the percentage of no RTW was 57.3%. The same pattern was observed when stratifying into WFNS grades (Table 2). Although no RTW was the lowest for those who had worked within the office, teaching, art, and finance sector in all WFNS categories, there were differences within this employment sector in accordance with the WFNS grade: no RTW was significantly higher in WFNS 3 and 4 patients than WFNS 1, 2, and 5 patients.

#### 3.2.3 Predictors for no return to work

Table 3 shows the results of the univariate and multivariate regression analyses for predictors of no RTW. There were many significant positive predictors in the univariate analysis, including age at ictus, clinically significant fatigue, WFNS grade, rebleed, large amounts of subarachnoid, intraventricular and intra-parenchymal blood, active smoking, severe multi-vessel spasm, CSF-shunting, DCI, length of invasive respiratory support, LOS, and the type of work at ictus. The sole protective predictor was higher education.

**Table 3:** Univariate and multivariate analyses of predictors for no return to work.

	**Univariate**			**Multivariate**		
	**OR**	**95% CI**	***P*** =	**OR**	**95% CI**	***p*** =
Women	1.017	0.635–1.627	0.945			
Age at ictus (years)	**1.045**	**1.018–1.072**	**< 0.001**	1.081	1.037–1.127	< 0.001
WFNS ref: WFNS1						
WFNS2	**2.109**	**1.139–3.905**	**0.018**			
WFNS3	**10.545**	**2.916–38.130**	**< 0.001**	8.003	1.871–34.229	0.005
WFNS4	**3.559**	**1.719–7.370**	**< 0.001**			
WFNS5	**2.966**	**1.476–5.960**	**0.002**			
Clinical fatigue (FSS ≥ 4.00)	**4.178**	**2.350–7.426**	**< 0.001**	5.576	2.515–12.360	< 0.001
Education (years)	**0.78**	**0.705–0.864**	**< 0.001**	0.754	0.646–0.879	< 0.001
Rebleed	**2.549**	**1.072–6.060**	**0.034**			
Smoking status ref: never						
Former	1.008	0.470–2.166	0.983			
active	**2.017**	**1.185–3.434**	**0.01**	2.335	1.156–4.717	0.018
ICH ref: none						
< 2 cm	0.911	0.388–2.319	0.831			
2–5 cm	**2.319**	**1.061–5.068**	**0.035**			
>5 cm	**6.378**	**1.796–22.652**	**0.004**			
Vasospasm ref: none						
Moderate in 1 vessel	1.375	0.734–2.576	0.321			
Moderate in multiple vessels	1.239	0.659–2.329	0.506			
Severe in 1 vessel	0.733	0.286–1.877	0.517			
Severe in multiple vessels	**2.27**	**1.052–4.898**	**0.037**			
Clinical vasospasm	1.558	0.895–2.714	0.115			
Delayed cerebral ischemia DCI	**2.526**	**1.274–5.077**	**0.008**			
Shunt	**2.738**	**1.532–4.895**	**< 0.001**			
Endovascular repair	1.007	0.638–1.587	0.978			
Intraventricular blood, LeRoux	**1.15**	**1.062–1.244**	**< 0.001**			
Modified Fisher, ref: 1						
2	**2.727**	**0.971–7.658**	**0.057**			
3	**2.784**	**1.659–4.672**	**< 0.001**			
4	**2.79**	**1.261–6.174**	**0.011**			
Time on respiratory support	**1.004**	**1.002–1.006**	**< 0.001**			
Length of stay	**1.043**	**1.013–1.075**	**0.005**			
Type of work, ref: office, teaching, and art sector						
Health and social sector	**1.936**	**1.057–3.547**	**0.032**			
Manufacture, construction, farming, and retail sector	**2.255**	**1.330–3.826**	**0.003**			

Variables entered into the multivariate analysis are in bold.

Higher education was demonstrated to be an independent protective predictor of no RTW in the multivariate analysis (Table 3, right columns). The independent predictors of no RTW were age at ictus, WFNS grade 3, and active smoking. The strongest independent predictor was the presence of clinically significant fatigue, which had a 5-fold risk of not returning to work. Excluding patients who were ≥62 years yielded the same results (age at ictus: OR: 1.055, 95% CI: 1.001–1.111, *p* = 0.046; WFNS3: OR: 29.084, 95% CI: 2.989–283.003, *p* = 0.004; active smoker: OR: 3.681, 95% CI: 1.540–8.799, *p* = 0.003; clinical fatigue: OR: 11.269, 95% CI: 3.833–33.135, *p* < 0.001; and higher education: OR: 0.782, 95% CI: 0.656–0.933, *p* = 0.006). When only including patients ≤ 55 years at the time of the hemorrhage also identified former employment within the manufacture, construction, farming, and retail as an independent predictor of no RTW (OR: 3.638, 95% CI: 1.251–10.579, *p* = 0.018). In that model, higher education and age were no longer predictors of no RTW, whereas both WFNS 3 and WFNS 5 became significant predictors (OR: 45.527, 95% CI: 4.617–448.986, *p* = 0.001 and OR: 2.275, 95% CI: 1.770–29.892, *p* = 0.006). Active smoking remained a significant predictor with OR 3.912, 95% CI 1.368–11.183, *p* = 0.011. Fatigue remained the strongest predictor with OR: 13.867, 95% CI: 3.691–52.098, *p* < 0.001.

When substituting WFNS with the Hunt and Hess Scale (38), the multivariate analysis identified the same independent predictors of no RTW apart from the fact that aSAH severity was no longer a significant predictor, whereas, ICH >2 cm became a significant predictor (OR 2.813, 95% CI 1.048–7.551, *p* = 0.040).

## 4 Discussion

### 4.1 Working capacity after aSAH

The ability to return to work may be considered the acid test of real-world functioning after a severe illness. In our aSAH patients, we found that return to gainful employment was 51.2%, with complete RTW accounting for 32.4%. These numbers concur with some studies (4, 15, 17, 21, 29, 39, 40) but are at the lower end when compared to the review by Al-Khindi et al., who found that most studies report an RTW of ~60% (5). The same study, however, highlights that RTW may be to jobs with less responsibility and/or fewer working hours and that RTW may be as low as 6–17% considering full RTW to pre-ictal level (5). Taking into account our fraction of partial RTW, our results align well with what is reported in the literature. Some of the inconsistencies in reported rates for RTW may be due to many studies evaluating RTW as a binary variable and not taking into account the working capacity or content of work performed, whereas other studies distinguish partial from complete RTW. This finding complicates the comparison of results and was also indicated in a review article investigating psychosocial comorbidities related to working capacity after aSAH (18). Most studies report rates of RTW at a fixed time-point of follow-up or when gainful employment was initiated, whereas we scored the time it took to reach the known maximum working capacity. This approach may explain why our time to work was much longer, with a median of 11 months than the 16 weeks reported by Gerner et al. (41). However, our results would be comparable to the studies examining RTW 1 year after the ictus (14, 26, 30). The time to initiate work and reach maximum capacity will also be different between countries in accordance with their welfare benefits and the availability of work reintegration programs. In Norway, the period of sick leave and work assessment allowance with economic compensation of at least 66% of premorbid income is relatively long, up to 5 years.

There is limited information about the increase in employment after aSAH, but we found that ~10% of our patients increased their work capacity after the hemorrhage. It is worth mentioning that none of them was in WFNS 3–5 grades. Quinn et al. reported that one of their patients (1.3%) who was not previously employed was actually full-time employed at follow-up after the aSAH (14).

### 4.2 Return to work and fatigue

Fatigue is a common, often long-lasting sequel that is present in approximately 70% of aSAH survivors when assessed with the FSS (11). There is a strong association between post-aSAH/stroke fatigue, mood disorders, and reduced quality of life, whereas the link to cognitive function is not very clear (9, 23), i.e., the subjective perception of fatigue seems to be more closely related to depression than to the objective, measurable cognitive performance (23, 42). On the other hand, RTW has been linked to cognitive function expressed as a psychomotor slowing (7), and one could argue that there may be an overlapping relation between fatigue, psychomotor speed, depressive symptoms, and no RTW. The statement that clinically significant fatigue has a negative impact on the ability to RTW (23) is confirmed by our present findings.

The inability to RTW has been linked to cognitive deficits (17, 21). Speech deficits (14) and left medial basal frontal as well as left lateral infarctions 1 year after the hemorrhage had a negative impact on the ability to RTW (10). This result could corroborate our finding of less RTW in the office, teaching, art, and finance sector, which may require the most cognitive and language ability to resume work at the same level. Psychomotor slowing and impaired delayed recall have been found to be a significant contributor to negative employment status after aSAH (7, 17, 21). Furthermore, neuropsychological tests assessing complex attention and executive functions could reliably distinguish between complete and incomplete RTW (6). Cognitive deficits also have an impact on the quality of work performed (43). Emotional problems, such as depression and anxiety, have also been identified as an independent predictor of unemployment from 6 months up to 10 years after aSAH (4, 14, 17–20). This finding indicates that emotional problems, especially depressive symptoms, not only have a negative impact on the ability to RTW but also represent a long-lasting problem (20). In fact, good outcome aSAH patients can feel not completely restituted and experience reintegration difficulties more than 20 years after their hemorrhage (44). Addressing emotional problems after aSAH more rigorously with pharmaceutical intervention and/or psychotherapy may not only have a positive effect on the quality of life but also on work status.

How patients themselves perceive their ability to perform at the same work level may be an important factor (45). Vilkki et al. observed that the patients' and their partners' subjective assessment of working and social ability was more closely linked to RTW than their actual more objective cognitive test results (10). Many patients also have unrealistic expectations regarding their recovery, which can negatively affect their rehabilitation progress (45). This impact is in part due to aSAH survivors having an unmet need for information (46). It should be the task of their specialist team to fill that information gap and provide realistic prospects for the recovery process (45, 46). A perceived failure of performance may be linked to fatigue. Patients' perception of recovery after aSAH was negatively affected by mental fatigue (45).

Return to work after aSAH seems to be determined more by the presence of chronic post-aSAH fatigue than the severity of hemorrhage *per se*. This corroborates with the findings by Gaastra et al. (22), who found that fatigue was a significant contributor to unemployment after aSAH, mediating as much as 24% effect on the latter. In their study, fatigue was not linked to LOS, which they used as a surrogate marker of aSAH severity (22). This finding is in line with our finding of a lack of agreement between frequency and intensity of fatigue in relation to WFNS grade. Employment is a multifaceted construct determined by an array of interrelated factors such as education and employment opportunities, age, social welfare programs, driving cessation, family constellations, personal factors, coping capabilities, and emotional problems, all of which again can be intertwined with chronic fatigue (1, 45, 47).

Smoking is related to socioeconomic factors (48), emotional problems (49), and maladaptive coping styles (50). Smoking may be a surrogate marker of these factors, which may explain why active smoking proves to be an independent predictor of no RTW in the present study. Furthermore, active smokers have higher levels of fatigue (11, 51). Our findings suggest that fatigue, in particular debilitating fatigue, needs to be addressed as an important element on the path to return to gainful employment.

### 4.3 Return to work in relation to aneurysm repair and aSAH complications

Al-Khindi et al. argued that cognitive deficits after aSAH are not strictly linked to hemorrhage severity but rather to complications such as vasospasm with DCI and/or hydrocephalus (5). SAH patients treated for acute hydrocephalus had a quadruple risk of only partial and not complete return to work as compared to those that had no cerebrospinal fluid diversion (6). Similarly, both aSAH-induced epilepsy and treatment with a cerebrospinal fluid shunt for post-hemorrhagic hydrocephalus are predictors of no RTW (31, 41). Presently, we identified severe vasospasm, DCI, and chronic hydrocephalus (shunt) as predictors of no RTW in the univariate analysis. The mode of aneurysm repair had no impact on RTW in our study, which aligns with other studies that found no difference in RTW between patients who underwent endovascular vs. surgical aneurysm repair (31, 52).

### 4.4 Return to work in relation to gender and education

Earlier studies found RTW to be lower in women (17, 25, 30, 41), whereas gender was no predictor of RTW in our study. In fact, more of our female participants had complete RTW rates than their male counterparts, although the difference was not significant. Interestingly, nine out of the ten patients who worked more after their hemorrhage than before were women. The discrepancy between our findings and that in the literature may be explained by higher female participation in the workforce in Norway (61% vs. ~40–45%) than in the said studies at the relevant time (53). Furthermore, our female participants had significantly more years of education, which was a positive predictor of RTW. This relation corroborates with long-term follow-up studies that found that those unemployed up to 13 years after SAH had fewer years of education (17, 20). Higher educational level has also been found to be related to better cognitive performance after aSAH (54), which in turn is related to employment status (5, 7). Those with education of ≥12 years have less global cognitive impairment than those with less education, and they are also more likely to improve their cognitive performance from 3 to 12 months after the hemorrhage (40). A lower level of education has been found to be a predictor of poor psychosocial quality of life 1 year after aSAH, and among those categorized into that group, as many as 91% were unable to fully resume their pre-ictal work status (15). Individuals without a college degree may not only be impeded by their health status after aSAH but may also have larger difficulties entering the job market in general. Hence, vocational training has been proposed as a means to increase job opportunities after aSAH in those with lower education (32).

### 4.5 Return to work and age

Similar to our findings, multiple studies report age as a dominant factor for employment status (14, 17, 19, 20, 31, 41). Hence, older individuals are more likely to not return to work at 6–12 months past the ictus (19), and this finding seems to be even more pronounced when looking at long-term outcomes over several years (20). Individuals who are close to pensionable age at their hemorrhage may choose to retire even though they could have been actually capable of returning to work (20). This finding is also illustrated by the flow of employment status in our study. Possibilities for retirement and permanent disability benefits will, though, be dependent on a country's welfare system. Hence, for long-term employment status, quality of life evaluations, fatigue, socioeconomic factors, and cognitive performance may be better indicators than age alone (20).

### 4.6 Return to work and aSAH severity

It is striking how inconsistent the literature is regarding RTW in relation to aSAH severity. We found that RTW was clearly higher in patients who presented low-grade aSAH (WFNS 1–2) as compared to those in WFNS 3–5, which corroborates with the findings of Seule et al. who reported work recovery (complete and partial) of 39% in WFNS 4–5 patients vs. 69% in WFNS 1–3 (16). This result is also consistent with the findings of Gerner et al. (41) and Nishino et al., even though the latter reported restricted patient inclusion to those aged 40–49 years at ictus and had no WFNS 5 patients included at follow-up (30). Another study also found aSAH severity to be a predictor of RTW 6 months after aSAH, but it included only very few poor-grade patients and identified emotional problems as the strongest predictor for no RTW (4). Taufique et al. found a poorer physical and psychosocial quality of life 1 year after aSAH presenting in Hunt and Hess grades 3 or higher, which, in turn, was related to lower odds of returning to previous employment levels (15). Others found no relation between aSAH severity and RTW (17, 21, 24, 32), with some of these studies having included a large fraction of WFNS 4–5 patients (17, 21). However, there seems to be no linear relationship between the inability to RTW and aSAH severity.

Recently, no RTW was the highest in WFNS grades 4 and 3, with the latter subgroup being a significant predictor of no RTW, i.e., patients presenting in WFNS grade 5 had better odds of gainful return to employment than those in WFNS grades 4 and 3. This finding may be linked to patients in WFNS 3–5 being more likely to have had larger intracerebral hematomas (ICH), which in turn could cause a neurological motor deficit. For WFNS 3, a motor deficit at ictus is a scoring criterion, and in some of these patients, the deficit may have become permanent.

When we looked at RTW in relation to Hunt and Hess grade, in fact, an ICH > 2 cm was a significant predictor of no RTW. Following this line of reasoning, the presence of ICH > 2 cm, but especially > 5 cm, was a significant predictor of no RTW in our univariate analysis regarding WFNS grades but did not reach significance in the multivariate analysis. Similarly, severe multi-vessel spasms and delayed cerebral ischemia (DCI) can cause permanent deficits, and both were significant predictors of no RTW in the univariate analysis. In accordance with the notion of parenchymal damage having an impact on the ability to RTW, residual physical disability has been found to predispose to no RTW (14). Passier et al. found no difference in life satisfaction across aSAH severity in terms of Hunt and Hess grades (29). On the other hand, life satisfaction was strongly linked to disability at discharge and RTW (29), which further strengthens the concept that neurological motor deficits are linked closer to no RTW than aSAH severity *per se*. Considering aSAH survivors with permanent neurological deficits, these would predominantly be from WFNS 3–4, as grade 5 patients with large hematomas would be more likely to succumb to their bleeding. Mortality in grade 5 patients is particularly high with 49–66%; however, the rate of survivors in a poor functional state is relatively lower than that in patients with ischemic middle cerebral artery infarctions, which would be comparable to those with large hematomas in the same area (55, 56).

### 4.7 Limitations

Our study has several limitations. We have not gathered information about mood disturbances, cognitive deficits, or socioeconomic factors beyond education level and the type of occupation in our patients. With such additional information, we could have gleaned a broader understanding of the interaction of these factors. Patients did not have an unlimited follow-up, and even though their work status may have been apprehended as final, it could have changed at a later point in time. This finding applies especially to those who made a full return to work and whose follow-up was terminated; they would have stepped down their workload at a later point in time. The follow-up regime differed between patients and did not follow a fixed scheme beyond 1 year. External validity is limited by the study being a single-center registry study and by the welfare benefits in our country. Fatigue was only measured with the FSS, which does not acknowledge all facets of fatigue. On the other hand, the FSS is one of the few fatigue questionnaires that are validated. The FSS evaluates the impact of fatigue on daily living, and one of the nine items directly asks about fatigue interfering with duties and work so that some degree of interference can be assumed with the mean FSS score and RTW status. The FSS was only assessed once in the chronic state after aSAH (>12 months after the hemorrhage) and not necessarily at the time when patients worked at their maximum capacity. On the other hand, fatigue that exists beyond 1 year after aSAH seems to remain at relatively stable levels over many years (11). Finally, we have not controlled for newly acquired concomitant diseases that could interfere with work status.

## 5 Conclusion

In the modern era of aSAH management, return to gainful employment is as low as 51.2%, with complete RTW accounting for 32.4%. The age at ictus is an independent predictor of no RTW but should be interpreted in the context of age categories of those close to retirement age vs. younger individuals. Clinically significant fatigue is the strongest independent predictor, accounting for a 5-fold risk of no RTW. Higher education is an independent protective predictor of no RTW. The mode of aneurysm repair and gender are not associated with regard to RTW. Patients in WFNS grades 1–2 more often returned to work than those in WFNS grades 3–5, but our results may indicate that neurological motor deficits are linked closer to no RTW than aSAH severity *per se*. Fatigue needs to be addressed as an important element on the path to return to gainful employment.

## Data availability statement

The raw data supporting the conclusions of this article will be made available by the authors, without undue reservation.

## Ethics statement

Ethical approval was not required for the study involving humans in accordance with the local legislation and institutional requirements. Written informed consent to participate in this study was not required from the participants or the participants' legal guardians/next of kin in accordance with the national legislation and the institutional requirements.

## Author contributions

AS: Conceptualization, Formal analysis, Funding acquisition, Investigation, Methodology, Supervision, Writing—original draft, Writing—review & editing. AL: Conceptualization, Writing—original draft, Writing—review & editing. EW: Conceptualization, Investigation, Writing—original draft, Writing—review & editing.
